# Induction and maintenance of a phenotypically heterogeneous lung tissue-resident CD4^+^ T cell population following BCG immunisation

**DOI:** 10.1016/j.vaccine.2018.07.035

**Published:** 2018-09-05

**Authors:** Naomi C. Bull, Daryan A. Kaveh, M.C. Garcia-Pelayo, Elena Stylianou, Helen McShane, Philip J. Hogarth

**Affiliations:** a Vaccine Immunology Team, Department of Bacteriology, Animal & Plant Health Agency (APHA), Addlestone, Surrey KT15 3NB, UK; b The Jenner Institute, University of Oxford, Old Road Campus Research Building, Roosevelt Drive, Oxford OX3 7DQ, UK

**Keywords:** BCG, Immunogenicity, Tissue-resident, T cell, Tuberculosis, Vaccine

## Abstract

Tuberculosis (TB) is the biggest cause of human mortality from an infectious disease. The only vaccine currently available, bacille Calmette-Guérin (BCG), demonstrates some protection against disseminated disease in childhood but very variable efficacy against pulmonary disease in adults. A greater understanding of protective host immune responses is required in order to aid the development of improved vaccines. Tissue-resident memory T cells (T_RM_) are a recently-identified subset of T cells which may represent an important component of protective immunity to TB. Here, we demonstrate that intradermal BCG vaccination induces a population of antigen-specific CD4^+^ T cells within the lung parenchyma which persist for >12 months post-vaccination. Comprehensive flow cytometric analysis reveals this population is phenotypically and functionally heterogeneous, and shares characteristics with lung vascular and splenic CD4^+^ T cells. This underlines the importance of utilising the intravascular staining technique for definitive identification of tissue-resident T cells, and also suggests that these anatomically distinct cellular subsets are not necessarily permanently resident within a particular tissue compartment but can migrate between compartments. This lung parenchymal population merits further investigation as a critical component of a protective immune response against *Mycobacterium tuberculosis* (*M. tb*).

## 1 Introduction

Tuberculosis (TB), caused by infection with *Mycobacterium tuberculosis* (*M. tb*), presents a major challenge to global health, claiming 1.7 million lives in 2016 [1]. The only licensed vaccine against TB, bacille Calmette-Guérin (BCG), was developed almost a century ago [2]. When BCG is administered intradermally in early life, it is protective against disseminated forms of TB in childhood [3]. However, efficacy against pulmonary disease in adulthood, the most common form of TB disease, is very variable [4].

In murine models of TB, BCG provides significant protection against infection [5], [6], [7], [8]. Despite strong evidence supporting a critical role for CD4^+^ T cells producing interferon-gamma (IFN-γ) in this protection [8], [9], frequencies of *M. tb*-specific CD4^+^ T cells in the blood and lymphoid organs of humans and mice do not correlate with protection [10], [11]. Similarly, magnitude and frequency of vaccine-induced IFN-γ responses fail to predict protective immunity [10], [11], [12]. A better understanding of the underlying mechanisms of vaccine-mediated protection, and generation of T cell memory in response to vaccination, is critical to rational development of more efficacious vaccines.

Tissue-resident memory T cells (T_RM_), a recently-identified subset of memory T cells, may play an important role in protective immunity to TB. T_RM_ persist in non-lymphoid tissues without re-circulating through the body and are present locally at sites of infection in multiple different tissues, including the lungs [13], [14], [15], [16]. They are able to mount a rapid *in situ* response to pathogenic challenge and can coordinate recruitment of immune cells to tissue sites [16], [17], [18]. Development of an intravascular staining technique has enabled the study of T_RM_, allowing definitive discrimination between cells resident within the parenchyma of an organ and those present within the vasculature [16], [19], [20], [21].

Several studies have investigated CD4^+^ T_RM_ in the lungs within the context of *M. tb* infection [17], [22], [23], [24], but their induction following BCG vaccination has not been well-characterised. Connor et al. [25] suggest that BCG-induced protection depends on lymphocyte migration to the lungs and retention of lung memory CD4^+^ T cells. Perdomo et al. [26] describe a transient influx of CD4^+^ and CD8^+^ T cells into the parenchyma of the lung following intratracheal BCG vaccination. However, neither of these studies utilised the intravascular staining technique for definitive identification of tissue-resident cells. This is critical, as previous studies utilising intravascular staining reveal that >95% of CD4^+^ T cells and >99% of total lymphocytes isolated from naïve murine lung via standard methods were in fact present in the vasculature of the lung rather than the parenchyma [17], [21].

Other studies have employed the use of intravascular staining to investigate responses to novel TB vaccines [27], [28], [29], [30], [31]. Woodworth et al. [27] found that mice immunised with a subunit TB vaccine generated polyfunctional CD4^+^ T cells which preferentially localised to the parenchyma of *M. tb*-infected lungs upon adoptive transfer. Carpenter et al. [28] demonstrated that vaccination with mycobacterial peptides resulted in a secondary CD4^+^ T cell response against *M. tb* challenge, comprised of antigen-specific cells preferentially localising to the lung parenchyma. Both of these vaccines conferred protection against *M. tb* infection, highlighting the exciting potential role of this subset in protective immunity.

The phenotype of CD4^+^ T cells induced by BCG vaccination has been described by several studies [5], [32], [33], but it is unclear how these phenotypes are distributed in the parenchymal and vascular compartments of the lung, as no studies have separately identified these populations with respect to BCG vaccine-induced responses. It is now important to establish whether BCG-induced lung parenchymal cells exhibit a unique phenotype, identifying them as tissue-resident. A number of studies have used expression of CD69 to define tissue-residence in the lung [16], [19], [20], [34], [35]. However, it is unclear how reliably this identifies lung T_RM_ in the context of TB vaccination. It is also important to determine whether the phenotype of these cells provides further knowledge regarding their functional potential. Whilst T_RM_ have been shown to express high levels of CD44 and low levels of CD62L, in common with effector memory T cells (T_EM_) [36]; they also exhibit a unique transcriptional profile, different from that of other memory T cell subsets [37], which confirms their classification as a separate population.

Here, we utilise the intravascular staining technique to comprehensively characterise the development of an antigen-specific tissue-resident CD4^+^ T cell population over the course of 12 months following intradermal BCG vaccination. We determine that BCG induces a population of these cells which are still present in the lung parenchyma 12 months post-immunisation. They display phenotypic and functional heterogeneity, reinforcing the importance of the intravascular staining technique for their definitive identification in the absence of unique phenotypic markers of lung location.

## 2 Results

### 2.1 Frequency of CD4^+^ T cells is greater in the lung vasculature than parenchyma post-BCG or placebo immunisation

**Fig. 1:**
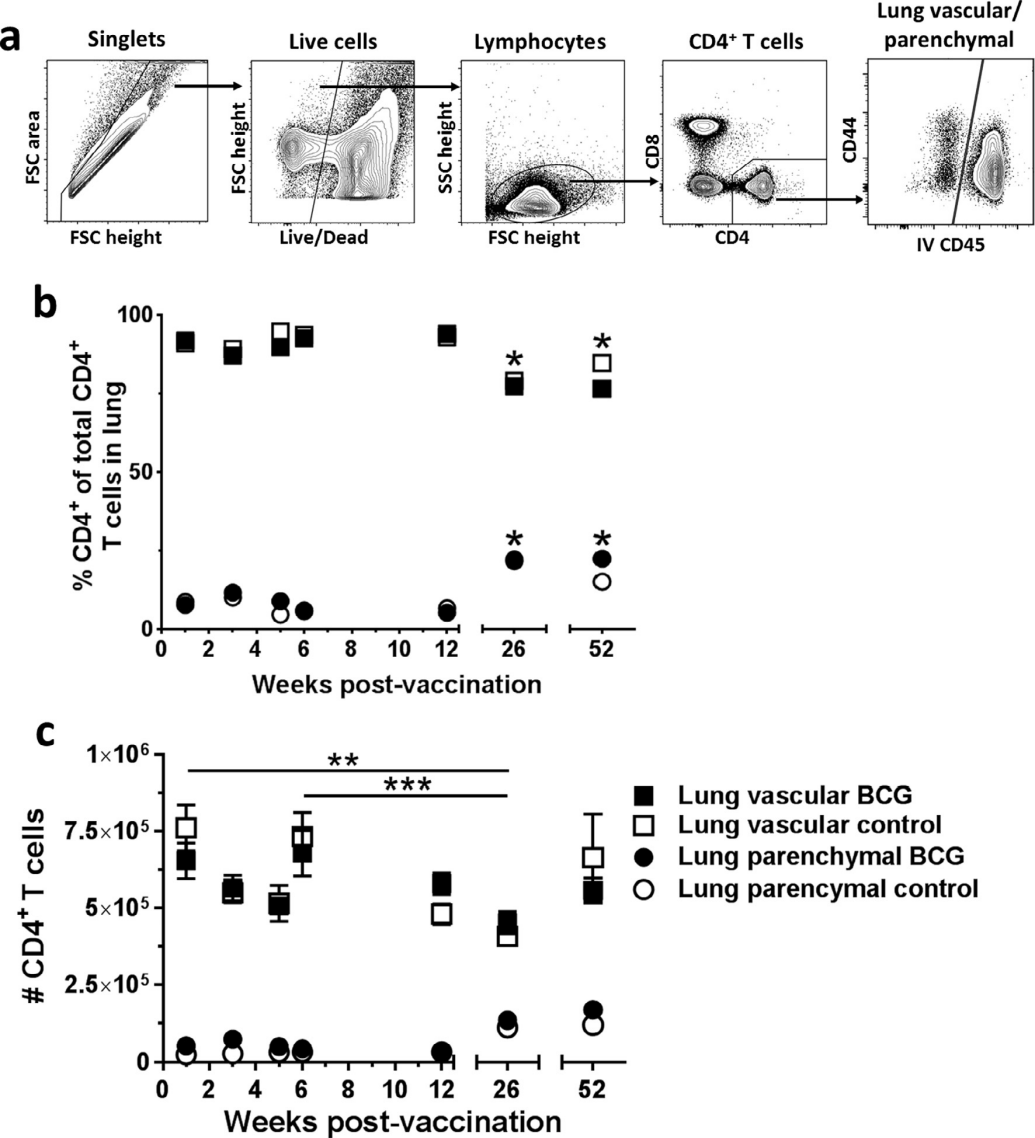
Frequency of CD4^+^ T cells is greater in the lung vasculature than parenchyma post-BCG or placebo immunisation. Following BCG immunisation, intravascular staining identified populations of lung parenchymal and lung vascular CD4^+^ T cells. (a) Representative plots from a BCG-immunised mouse showing gating strategy for defining lung parenchymal and vascular populations. (b) Frequency of lung vascular and parenchymal CD4^+^ T cells as a % of total CD4^+^ T cells isolated from the lung. (c) Number of CD4^+^ T cells in the lung parenchyma and vasculature. For both graphs, points represent mean ± SEM (*n* = 6). Two-way ANOVA with Sidak’s post-test, comparing each time point within the same compartment (shown on graph, ^*^P < 0.05, ^**^P < 0.01, ^***^P < 0.001), BCG with control (no significant differences at any time point) and lung parenchymal with lung vascular (not shown on graph, for all time points frequency and number of lung vascular CD4^+^ T cells exceeded lung parenchymal CD4^+^ T cells in both BCG-immunised and control mice by ^****^P < 0.0001).

Following intradermal vaccination with BCG or placebo, intravascular anti-CD45 staining allowed discrimination between CD4^+^ T cells present in the lung parenchymal tissue and those present in the lung vasculature (Fig. 1a). At all time points investigated, up to 12 months post-vaccination, frequencies and absolute numbers of CD4^+^ T cells in the lung vasculature were significantly higher than in the parenchyma, for both BCG and placebo-immunised mice (*P* < 0.0001) (Fig. 1b and c). For the first 12 weeks post-immunisation, frequencies of CD4^+^ T cells in the lung vasculature were approximately 9-fold greater than in the parenchyma. At 26 and 52 weeks post-immunisation, frequencies of CD4^+^ T cells in the lung parenchyma were significantly greater than for all previous time points (*P* < 0.05) and frequencies of CD4^+^ T cells in the lung vasculature were significantly lower than for all previous time points (*P* < 0.05). The actual number of CD4^+^ T cells in the lung parenchymal compartment did not alter significantly between any of the time points measured post-immunisation. There were significantly fewer CD4^+^ T cells in the lung vascular compartment at week 26 (4.5 × 10^5^) compared to weeks 1 (6.5 × 10^5^, *P* = 0.0022) and 6 (6.8 × 10^5^, *P* = 0.0004) in both BCG and placebo-immunised mice.

### 2.2 BCG vaccination induces antigen-specific CD4^+^ T cells in the lungs, spleen and blood

Supplementary data associated with this article can be found, in the online version, at https://doi.org/10.1016/j.vaccine.2018.07.035.

**Fig. 2:**
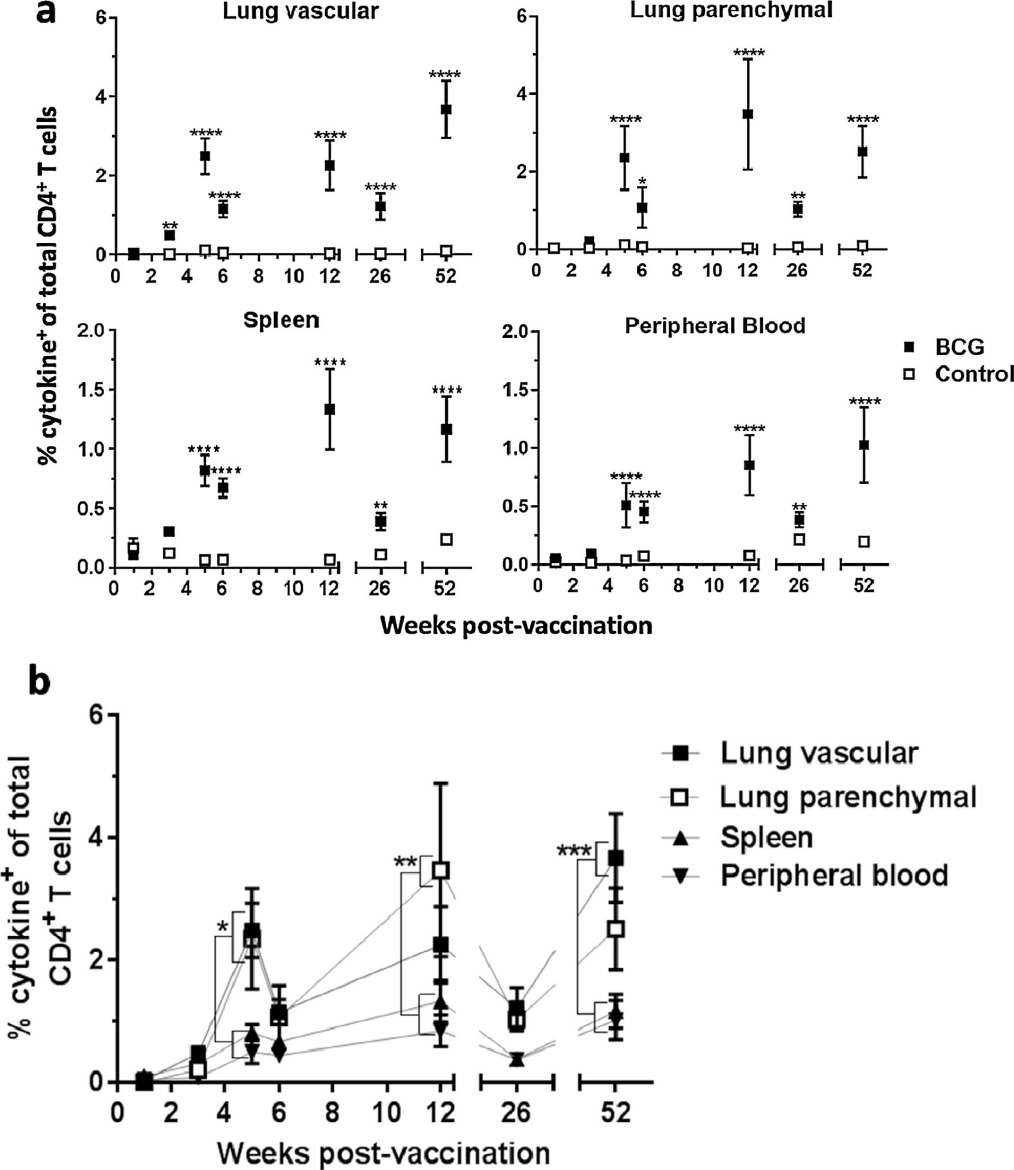
BCG induces antigen-specific CD4^+^ T cells in the lungs, spleen and peripheral blood, with highest frequencies in the lung parenchyma and vasculature. Following BCG immunisation, intravascular staining and ICS identified populations of antigen- (TB10.4 peptide) specific (cytokine^+^) CD4^+^ T cells in the lungs, spleen and peripheral blood producing IFN-γ, IL-2 or TNF-α alone or in combination. (a) Frequencies of antigen-specific CD4^+^ T cells as a % of the total number of CD4^+^ T cells in the same compartment. Statistical comparison is between BCG and control at each time point. (b) Frequencies of antigen-specific CD4^+^ T cells from all compartments in BCG-vaccinated animals expressed as a % of the total number of CD4^+^ T cells in the same compartment. Statistical comparison is between frequency of BCG-induced antigen-specific CD4^+^ T cells in all compartments at each time point. For all graphs, points represent mean ± SEM (*n* = 6). Two-way ANOVA with Sidak’s post-test, ^*^P < 0.05, ^**^P < 0.01, ^***^P < 0.001, ^****^P < 0.0001.

In order to investigate the development of antigen-specific CD4^+^ T cells following BCG vaccination, lymphocytes isolated from the lungs, spleen and peripheral blood were stimulated with a pool of TB10.4 peptides before intracellular cytokine staining (ICS) to identify CD4^+^ T cells producing interferon-gamma (IFN-γ), interleukin-2 (IL-2) and tumour necrosis factor-alpha (TNF-α) (Supplementary Fig. 1). Boolean gating allowed analysis of all CD4^+^ T cells producing any of these cytokines independently or in combination (cytokine^+^). BCG vaccination induced significant populations of antigen-specific CD4^+^ T cells in the lung, spleen and peripheral blood, compared to placebo immunisation (*P* < 0.05) (Fig. 2a). Antigen-specific CD4^+^ T cells were identified at all time points from week 3 post-BCG vaccination in the lung vasculature and from week 5 post-BCG vaccination in the lung parenchyma, spleen and peripheral blood.

**Supplementary material:**

### 2.3 BCG induces the highest frequencies of antigen-specific CD4^+^ T cells in the lung, with no difference in frequency between parenchyma and vasculature

There was no significant difference in frequency of antigen-specific CD4^+^ T cells present in the lung vascular and parenchymal compartments at any time point post-BCG vaccination (Fig. 2b). At week 5 post-vaccination, both the lung parenchyma and vasculature contained significantly higher frequencies of antigen-specific CD4^+^ T cells (2.36% and 2.49% respectively) when compared to peripheral blood (0.51%, *P* = 0.0146 & *P* = 0.0074 respectively). The lung vascular compartment contained significantly higher frequencies of antigen-specific CD4^+^ T cells when compared to spleen (0.82%, *P* = 0.0338). At week 12 post-vaccination, only the lung parenchymal compartment contained significantly higher frequencies of antigen-specific CD4^+^ T cells (3.48%) compared to spleen (1.34%, *P* = 0.0055) and peripheral blood (0.85%, *P* = 0.0002). At week 52 post-vaccination, only the lung vascular compartment contained significantly higher frequencies of antigen-specific CD4^+^ T cells (3.68%) compared to spleen (1.17%, *P* = 0.0004) and peripheral blood (1.03%, *P* = 0.0001).

### 2.4 BCG-induced antigen-specific CD4^+^ T cells display a dominance of multifunctional cells in the lungs, spleen and blood

**Fig. 3:**
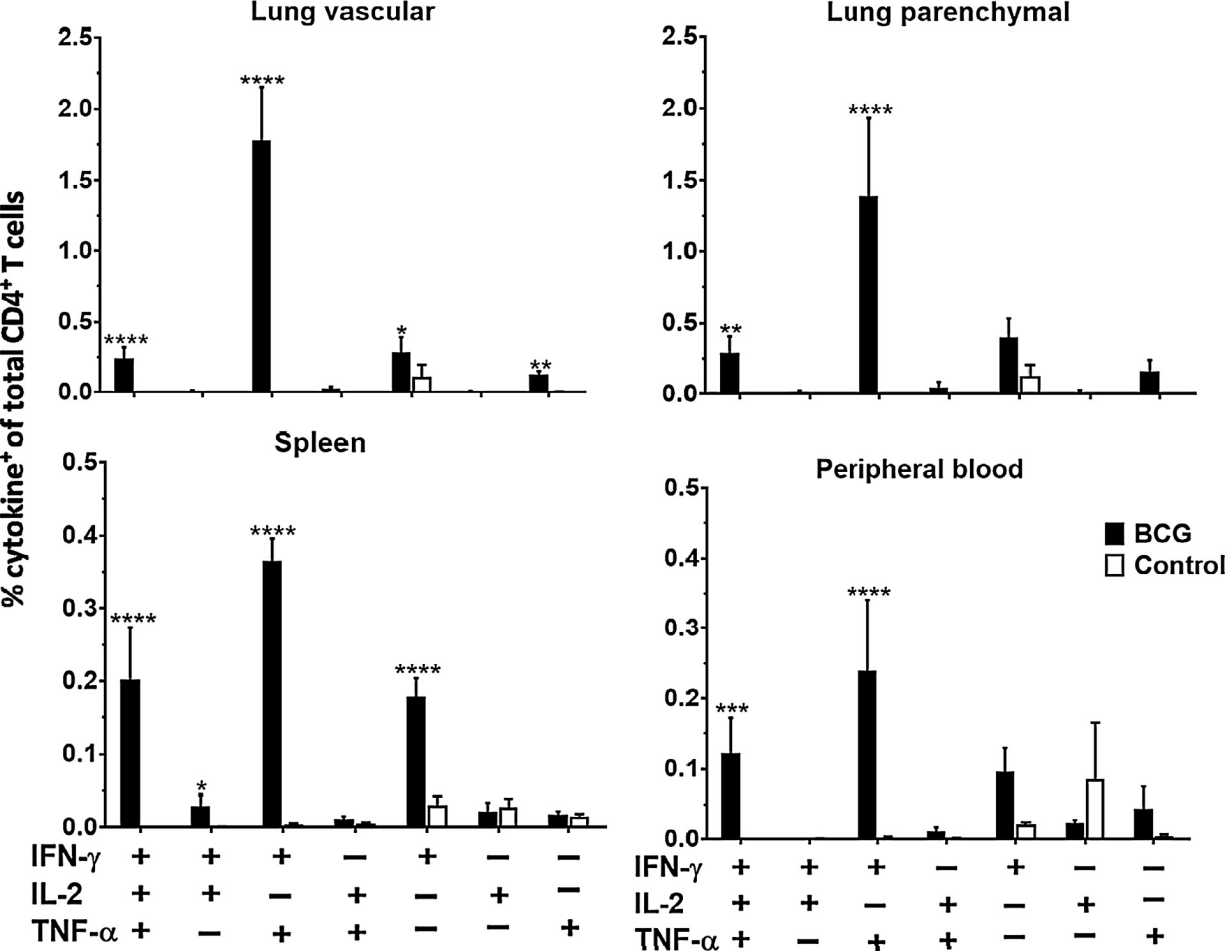
BCG-induced antigen-specific CD4^+^ T cells display a dominance of multifunctional cells. Following BCG immunisation, intravascular staining and ICS identified populations of antigen-specific (cytokine^+^) CD4^+^ T cells in the lungs, spleen and peripheral blood producing IFN-γ, IL-2 or TNF-α alone or in combination. Graphs show frequencies of BCG-induced antigen-specific CD4^+^ T cells in all compartments as a % of the total CD4^+^ T cells in the same compartment at week 5 post-BCG vaccination. Data for all other time points is presented in Supplementary Fig. 2. For all graphs, bars represent mean ± SEM (*n* = 6). Two-way ANOVA with Sidak’s post-test, comparing BCG and control, ^*^P < 0.05, ^**^P < 0.01, ^***^P < 0.001, ^****^P < 0.0001.

Boolean gating analysis was used to identify populations of antigen-specific CD4^+^ T cells producing IFN-γ, IL-2, TNF-α or any combination of the three. Triple-positive (IFNγ^+^IL-2^+^TNF-α^+^) and double-positive (IFNγ^+^IL-2^−^TNF-α^+^) CD4^+^ T cells were detectable in all compartments at all time points post-BCG vaccination except week 1, when there were no significant populations of antigen-specific CD4^+^ T cells in any compartment. Representative data for week 5 is shown (Fig. 3), with data for all other time points available in Supplementary Fig. 2. Similar patterns of cytokine production were seen across all time points.

### 2.5 BCG-induced antigen-specific CD4^+^ T cells display an effector phenotype in the lungs, spleen and blood

**Fig. 4:**
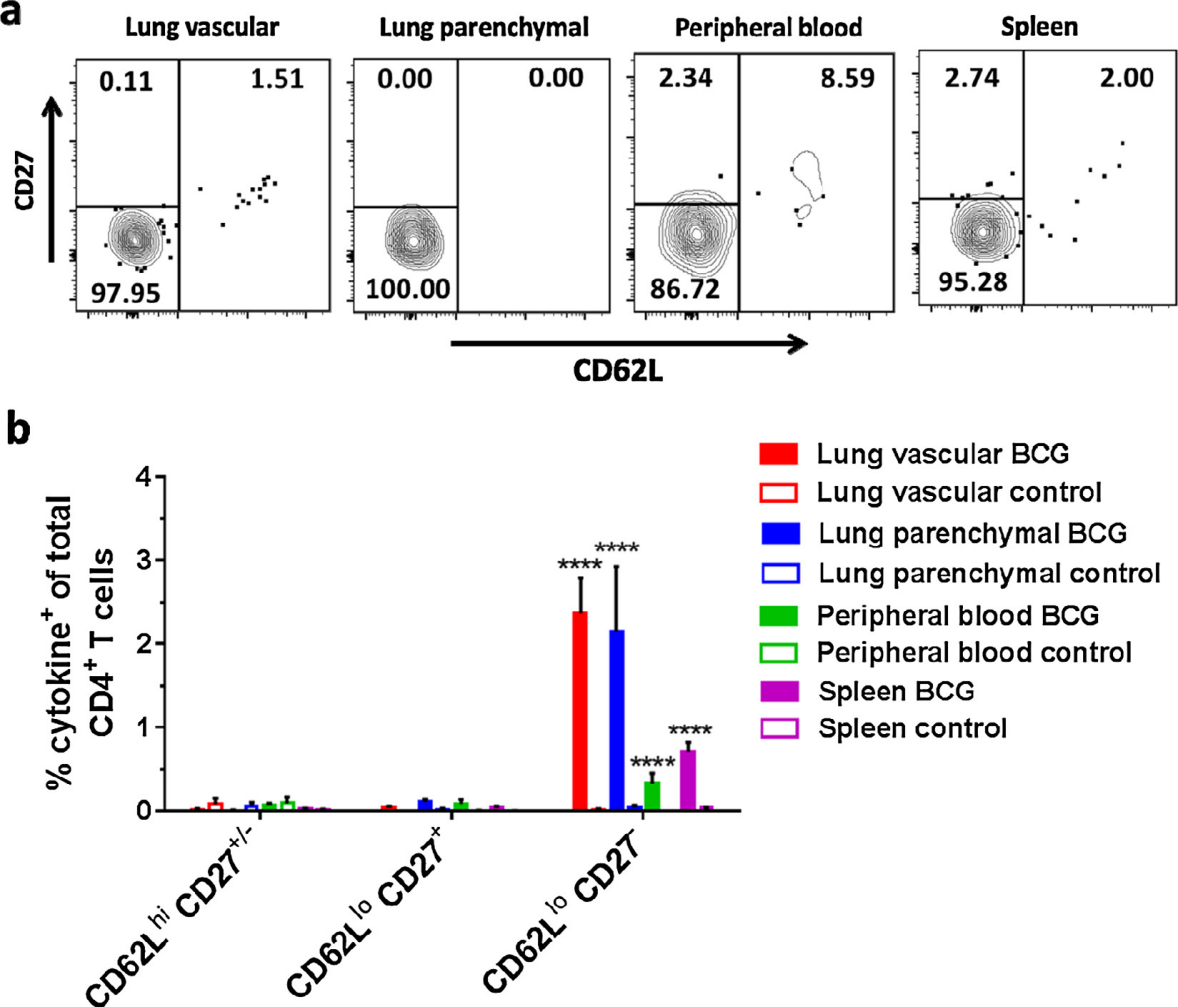
BCG-induced antigen-specific CD4^+^ T cells display an effector phenotype. Following BCG immunisation, intravascular staining and ICS identified populations of antigen-specific (cytokine^+^) CD4^+^ T cells. (a) Representative plots showing surface staining for CD62L and CD27 on antigen-specific CD4^+^ T cells from a mouse 5 weeks post-BCG vaccination. (b) Frequency of antigen-specific CD4^+^ CD44^hi^ T cells in all compartments displaying combinations of CD62L and CD27 cell surface markers as a % of total CD4^+^ T cells in that compartment at week 5 post-vaccination. Data for all other time points is presented in Supplementary Fig. 3. Bars represent mean ± SEM (*n* = 6). Two-way ANOVA with Sidak’s post-test, comparing BCG and control, ^****^P < 0.0001.

In order to determine whether the antigen-specific CD4^+^ T cell populations induced by BCG vaccination displayed an effector phenotype, expression of CD62L was investigated [38] (Fig. 4a). CD27 expression was investigated as a marker of functional heterogeneity in CD4^+^ memory T cells [39], [40] (Fig. 4a). Prior to analysis of CD62L and CD27 expression, CD4^+^ T cells were pre-gated on CD44^hi^ to identify antigen-experience [41] (Fig. 1a). At all time points from week 3 post-BCG vaccination, antigen-specific CD4^+^ T cells in both lung compartments, spleen and peripheral blood all displayed a CD44^hi^ CD62L^lo^ CD27^−^ effector phenotype (*P* < 0.0001). Representative data for week 5 is shown (Fig. 4b) with data for all other time points available in Supplementary Fig. 3.

### 2.6 BCG-induced antigen-specific CD4^+^ T cells are CCR7^−^ but heterogeneous for CD69 and CD127

**Fig. 5:**
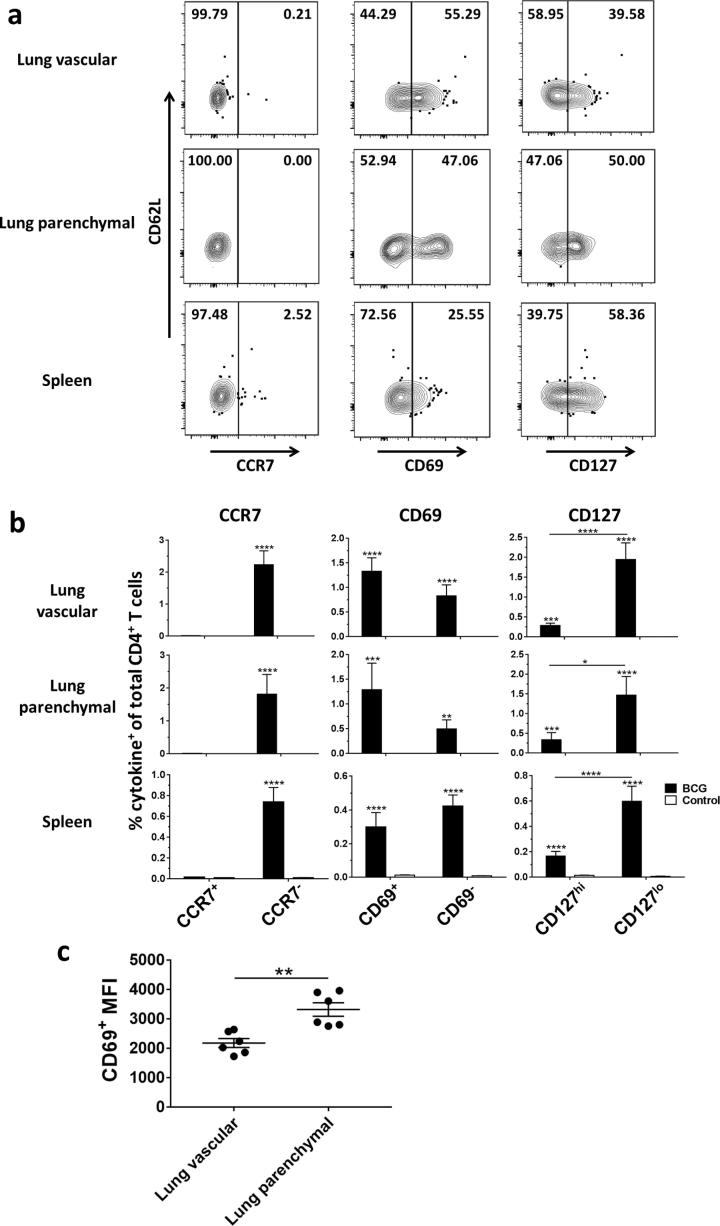
BCG-induced antigen-specific CD4^+^ T cells are CCR7^−^ but heterogeneous for CD69 and CD127. Following BCG immunisation, intravascular staining and ICS identified populations of antigen-specific (cytokine^+^) CD4^+^ T cells. (a) Representative plots showing surface staining for CCR7, CD69 and CD127 on antigen-specific CD4^+^ T cells from a mouse 5 weeks post-BCG vaccination. (b) Frequency of antigen-specific CD4^+^ CD62L^lo^ T cells in all compartments displaying CCR7, CD69 and CD127 cell surface markers as a % of total CD4^+^ T cells in that compartment at week 5 post-BCG vaccination. Data for all other time points is represented in Supplementary Fig. 4. For all graphs, bars represent mean ± SEM (*n* = 6). Two-way ANOVA with Sidak’s post-test, comparing BCG with control and BCG with BCG, ^*^P < 0.05, ^**^P < 0.01, ^***^P < 0.001, ^****^P < 0.0001. (c) Median fluorescence intensity (MFI) of antigen-specific CD4^+^ CD69^+^ populations in the lung parenchyma and lung vasculature at week 5 post-vaccination. Data for all other time points is represented in Supplementary Fig. 5. Lines represent mean ± SEM (*n* = 6). Unpaired two-tailed *t*-test, ^**^P < 0.01.

Additional markers were investigated to further characterise the antigen-specific CD4^+^ T cells in each compartment. We included CCR7 to define effector or central memory phenotype [38], CD127 to determine memory capability [42], [43] and CD69 as it has been suggested as a putative marker of T_RM_ [37], [44]. At all time points from week 3 post-BCG vaccination, antigen-specific CD4^+^ T cells in both lung compartments, spleen and peripheral blood were all CCR7^−^, either CD69^+^ or CD69^−^ and either CD127^hi^ or CD127^lo^ (Fig. 5a). Representative data for week 5 is shown (Fig. 5b) with data for all other time points available in Supplementary Fig. 4. There were significantly higher frequencies of CD127^lo^ compared to CD127^hi^ CD4^+^ T cells in both lung compartments and spleen at weeks 3, 5 and 6 (*P* < 0.05). There were significantly higher frequencies of CD69^+^ compared to CD69^−^ CD4^+^ T cells in both lung compartments and spleen at weeks 12 and 26 (*P* < 0.01) and in the lung vascular compartment alone at week 3 (*P* = 0.0497). There were significantly higher frequencies of CD69^−^ compared to CD69^+^ CD4^+^ T cells in the lung vascular compartment and the spleen at week 52 (P < 0.0001). In order to investigate differences in the level of CD69 expression on lung parenchymal and vascular CD4^+^ T cells, median fluorescence intensity (MFI) of the CD69^+^ populations was measured (Fig. 5c shows data for week 5 post-BCG and Supplementary Fig. 5 shows all other time points). The CD69^+^ MFI was significantly higher for lung parenchymal compared to lung vascular CD4^+^ T cells at weeks 5 (*P* < 0.0021) and 52 (*P* < 0.0032) post-BCG. At all other time points, the CD69^+^ MFI was higher for the lung parenchymal population, but this did not reach statistical significance.

## 3 Discussion

BCG vaccination provides significant protection against TB in mice [5], [6], [7], [8], but despite strong evidence implicating IFN-y-producing CD4^+^ T cells in this protection [8], [9], the precise underlying mechanisms have yet to be elucidated. T_RM_, a subset of memory T cells which persist in non-lymphoid tissues and are able to respond to infection locally at sites of pathogen entry, have been identified in the lungs within the context of *M. tb* infection [17], [21], [22], [24]. They have been shown to mediate superior protection, compared to T cells present in the lung vasculature [17], [22], [24], and their ability to enter the lung parenchyma correlates with greater control of *M. tb* [17], [22].

To date, there has been little data published on T_RM_ induction following immunisation against TB. Woodworth et al. [27] and Carpenter et al. [28] have both used intravascular staining to investigate CD4^+^ T cell responses to vaccination against TB, using subunit and peptide immunisations respectively. Both of these studies describe induction of a population of CD4^+^ T cells which preferentially localise to the parenchyma of *M. tb*-infected lungs upon adoptive transfer. Here we evaluate whether BCG, the only licensed vaccine currently available against TB, delivered intradermally, can induce a population of antigen-specific CD4^+^ T cells in the lung parenchyma. We describe the development of a lung tissue-resident CD4^+^ T cell population following BCG which persists for >12 months post-vaccination. In the murine model, protective immunity afforded by parenteral BCG immunisation has been demonstrated up to 12 months post-vaccination [32], [45]. The durability of this protection has been linked to the ongoing presence of BCG-specific, multifunctional CD4^+^ T cells [5], [45], and here we present the first study utilising intravascular staining to investigate their location in either the parenchyma or vasculature of the lung.

We identified BCG-induced antigen-specific CD4^+^ T cells in the lung parenchyma from 5 weeks post-vaccination, and this tissue-resident population was maintained for the duration of the study, 52 weeks in total. Factors involved in the maintenance of T_RM_ in the lung are incompletely understood, and no data is published on their persistence following vaccination against TB, utilising the intravascular staining technique. Longitudinal studies of CD8^+^ lung T_RM_ report waning of the population over time following clearance of influenza virus infection [35], [46]. Slϋtter et al. [46] suggest that this is due to a requirement for continual replenishment from the circulating memory T cell pool. Our results demonstrate maintenance of a population of tissue-resident antigen-specific CD4^+^ T cells up to 52-weeks post-infection, with no measurable decline. This difference may be due to the fact that BCG has been shown to persist in tissues for at least 16 months post-vaccination providing ongoing antigenic-stimulation [5], [47], resulting in maintenance of a stable tissue-resident population. Additionally, Turner et al. [48] report that in a murine model of allergic airway disease, only CD4^+^ and not CD8^+^ T_RM_ persisted long term in the lung following cessation of exposure to allergen. This suggests that the dynamics of CD4^+^ T_RM_ responses may well differ from those of their CD8^+^ counterparts.

Use of intravascular staining [21] allowed definitive identification of tissue-resident cells, whilst also enabling separate analysis of T cells present within the vasculature of the lung. Antigen-specific CD4^+^ T cells were present in the lung vasculature from 3 weeks post-vaccination, but were undetectable in the peripheral blood until 5 weeks post-vaccination, suggesting that the lung vascular compartment is not simply an extension of the systemic circulation but may be considered a distinct immunological compartment in its own right. This hypothesis is supported by several studies investigating the kinetics of migration of leukocytes through the vasculature of the lungs [49], [50], [51], [52], which indicate that leukocytes may be retained within the capillary bed of the lung whilst migrating. In accordance with this, Sakai et al. [17] demonstrated that during *M. tb* infection, the frequency of antigen-specific CD4^+^ T cells in the lung vasculature was >5-fold higher than in the peripheral circulation. In fact, the authors also report that these lung vascular CD4^+^ T cells produced more IFN-γ than their tissue-resident counterparts, suggesting that they may also have an important role to play in protection against *M. tb* infection.

Whilst an antigen-specific CD4^+^ T cell response was maintained in all compartments from week 5 post-BCG for the duration of the study, the magnitude of this response varied between the time points measured. Peak responses were observed at weeks 5, 12 and 52 post-vaccination, with lower frequencies of antigen-specific CD4^+^ T cells observed at time points in between these. We have observed this previously [5], [32] and it is therefore a consistent observation in our long term BCG vaccination studies in mice. We speculate this may be because BCG is a live, replicating vaccine undergoing cycles of control and subsequent replication within the host, resulting in changes in the magnitude of the antigen-specific response. Further work is required in order to test this hypothesis.

At all time points post-immunisation, the proportion of total lung CD4^+^ T cells present in the vasculature was significantly greater than the proportion present in the parenchyma. In spite of the relative larger size of the lung vascular population, from week 5 post-BCG vaccination the magnitude of the antigen-specific CD4^+^ T cell response in both lung compartments was comparable. This suggests that systemic BCG is equally capable of inducing tissue-resident and lung vascular responses. Notably, at week 5 post-vaccination, antigen-specific CD4^+^ T cells in both lung compartments were observed more frequently than in the peripheral blood. This supports evidence in the existing literature that systemic BCG vaccination is able to induce lung-specific responses [32].

By week 26 post-immunisation, the difference in proportion of total CD4^+^ T cells between both lung compartments was reduced, although there was still a significantly greater proportion of the total CD4^+^ T cell population present in the lung vasculature. However, this was the case for both BCG and placebo-immunised mice and therefore may reflect a physiological ageing change in the mice rather than a specific effect of BCG vaccination. Zens et al. [53] have recently reported impaired establishment of T_RM_ during infancy and suggest that this is due to intrinsic differences in infant and adult T cell populations. Further investigation is required, as this may have important implications for the timing of administration of vaccines in order to optimise generation of T_RM_.

We performed detailed cell-surface phenotype analysis, in order to identify unique phenotypic characteristics of the tissue-resident population induced following BCG vaccination. All antigen-specific CD4^+^ T cells, regardless of location, were CD44^hi^ CD62L^lo^ CD27^−^ as described previously [32]. Importantly, we found no significant differences between lung compartments in the proportion of CD4^+^ T cells expressing CD69. However, there did appear to be a relationship between higher expression of CD69 and location, as the MFI of CD69^+^ cells in the parenchyma was consistently higher than those in the vasculature at all time points measured, despite this only reaching statistical significance at weeks 5 and 52 post-vaccination. CD69 has been described as a putative marker of tissue-residence on T_RM_ [37], [44], [54] due to its ability to inhibit sphingosine-1-phosphate receptor 1 (S1PR1)-mediated T cell exit from secondary lymphoid organs [55], [56], [57]. This has been proposed as a mechanism for maintenance of T_RM_ in tissues [44], [58]; therefore, the observed higher CD69 expression on parenchymal cells may be related to their retention in the lung tissue. However, the finding that expression of CD69 alone does not differentiate between lung parenchymal and vascular T cells reinforces the need to utilise the intravascular staining technique to definitively identify lung parenchymal cells, as to date no unique phenotypic markers have yet been identified to describe tissue-resident cells.

Further analysis revealed antigen-specific CD4^+^ T cells in the lungs and spleen share a CD62L^lo^ CCR7^−^ effector memory T cell (T_EM_), rather than central memory (T_CM_) phenotype, consistent with previous findings [5], [38], [59]. Indeed, it has been suggested that the failure of BCG to provide durable long-term protection may be due to its inability to induce T_CM_ [59], [60]. In humans, generation of T_CM_ requires contraction of the BCG-specific effector T cell response [61]. Here, we have shown that T_EM_ responses are maintained at stable levels in the lung, spleen and peripheral blood of mice up to 12 months post-BCG vaccination, possibly due to persistence of BCG providing chronic antigenic stimulation [5], [47]. Studies suggest that BCG may persist for many years in humans [62], [63], [64], [65], [66], offering a possible explanation for the observed lack of development of T_CM_ following vaccination [67], [68], [69], and subsequent waning of immunity [70]. However, this theory is confounded by evidence of long-term BCG-mediated protection in some populations [71]. Further work is required to determine the features of BCG-induced T cell immunity which determine subsequent durability of protection.

Expression of CD127 (IL-7 receptor subunit-α) was of interest, as IL-7 signalling promotes long-term survival of memory CD4^+^ T cells, and is a key regulator during memory development [72], [73]. Therefore, expression of CD127 has been used as a marker of T cell memory capability [42], [43]. Significant populations of antigen-specific CD127^hi^ and CD127^lo^ CD4^+^ T cells were observed in both the parenchyma and the vasculature of the lungs, as well as the spleen and peripheral blood, at all time points post-BCG vaccination. These data suggest the simultaneous presence of both memory and effector T cell populations, mediated by chronic antigen exposure due to the persistence of BCG [5], [47].

Whilst the phenotyping data presented here give an interesting insight into the nature of the antigen-specific CD4^+^ T cell response induced in the lung parenchyma following BCG vaccination, interpretation of this data is confounded by use of a stimulation protocol to allow for identification of cytokine production. This may have impacted on phenotype analysis through uniform stimulation of all antigen-specific cells. Further work using tetramer staining to identify phenotype directly *ex vivo* will be required to confirm these findings. In addition, characterisation of the lung parenchymal population through expression of CXCR3, CX3CR1 and KLRG1 would also provide valuable information, as combinations of these markers have been utilised in other studies to define the lung parenchymal population following *M. tb* infection [17] and subunit TB vaccination [27].

Functional capacity was also assessed and across all tissue sites there was a dominance of multifunctional cells, producing two or more cytokines. No distinct differences in the pattern of cytokine production were evident between compartments.

This high level of heterogeneity, and the common phenotypic characteristics shared between BCG-induced CD4^+^ T cells in multiple locations, may suggest that these cells can migrate, rather than being maintained in distinct tissue compartments separately from one other. The lack of clear functional differences between CD4^+^ T cells in the lung vascular and parenchymal compartments may suggest that they are in fact the same subset of antigen-specific cells having undergone a process of extravasation. Interestingly, Woodworth et al. [27] recently described a population of circulating CD4^+^ T cells, induced by a subunit vaccine against TB, which shared phenotypic characteristics of lung parenchymal CD4^+^ T cells and efficiently trafficked into *M. tb*-infected lung parenchyma. This may indicate that it is not necessary for CD4^+^ T cells to be permanent residents in the lung in order for them to provide protection against *M. tb* infection. Indeed it may be enough for a vaccination to induce a population of CD4^+^ T cells with the characteristics of lung parenchymal cells, which are able to efficiently migrate to the lung when presented with a pathogenic challenge. This may have implications for the design of future vaccination strategies to improve upon protection afforded by BCG.

In conclusion, we demonstrate that BCG delivered systemically induces tissue-resident, antigen-specific CD4^+^ T cells in the lung parenchyma detectable up to 12 months post-vaccination. These cells are defined as tissue-resident through their location in the parenchyma, as identified through intravascular staining. This may represent a long-lived vaccine-induced T_RM_ population, situated within the lung tissue ready to respond in the event of infection with *M. tb*. Antigen-specific CD4^+^ T cells are also maintained in the lung vasculature, spleen and peripheral blood, confirming that BCG induces durable immune responses both locally and systemically. These memory responses are enriched in lung compartments compared to the spleen and peripheral blood, with high levels of heterogeneity found in all compartments. Within the scope of this study it was not possible to identify a unique tissue-resident immune signature. An increased understanding of the immune responses and protective mechanisms induced by BCG vaccination will contribute to rational development of more protective vaccination regimens.

## 4 Methods

### 4.1 Ethics

All animal work was carried out in accordance with the UK Animal (Scientific Procedures) Act 1986, under appropriate Personal and Project licences. The study protocol was approved by the APHA Animal Use Ethics Committee.

### 4.2 Animals

Female specific-pathogen-free (SPF) BALB/c mice were obtained from Charles River UK and used at 8 weeks of age. Animals were housed in appropriate biological containment facilities at APHA, according to the Code of Practice for the Housing and Care of Animals Bred, Supplied or Used for Scientific Purposes. All animals were randomly assigned to treatment groups, housed in groups of 6 mice per cage and provided food and water *ad libitum*. Provision of normally distributed data for immunological analyses required minimum sample size *n* = 6 (Kolmogorov and Smirnov test).

### 4.3 Immunisation

Mice were immunised with the human vaccine strain *M. bovis* BCG Danish prepared as per manufacturer’s instructions (SSI, Denmark). A single dose of 2 × 10^5^ colony forming units (CFU) of BCG in 50 µl inoculum was administered via intradermal injection in the base of the tail. Control mice received 50 µl Hank’s Balanced Salt Solution (HBSS) (Gibco) administered in the same way.

### 4.4 Intravascular stain

Intravascular staining was performed using an amendment of the method described by Anderson et al.[21] Briefly, 100 µl of PE-conjugated anti-CD45 monoclonal antibody (eBioscience) at 0.75 µg/ml in HBSS was administered via the lateral tail vein one minute prior to euthanasia, allowing flow-cytometric discrimination between lung vascular cells (accessible to the stain) and lung parenchymal cells (inaccessible to the stain).

### 4.5 Lymphocyte isolation

Spleen cells were isolated by passage through a 40 µm cell strainer, washed at 300 g for 8 min and resuspended at 1 × 10^7^ cells/ml in Dulbecco’s Modified Eagle’s Medium (DMEM) (Sigma) supplemented with foetal calf serum (FCS) and penicillin/streptomycin (Gibco) for assays.

Lung cells were isolated using a GentleMACs™ tissue dissociator and C tubes (Miltenyi Biotech). Cells were agitated for 1 h at 37 °C in supplemented DMEM with collagenase I (Gibco) and DNase II (Sigma), passed through a 40 µm cell strainer, washed and resuspended in supplemented DMEM at 5 × 10^6^ cells/ml for assays.

Peripheral blood cells were isolated through incubation for 10 min at room temperature with MACS rinsing buffer (Miltenyi Biotech) and anti-Terr119-biotin (eBioscience) at 6.7 µg/ml, followed by addition of EasySep™ mouse streptavidin RapidSpheres™ (STEMCELL Technologies Inc.) at 174 µl/ml and placement on EasySep™ magnet for 5 min. Supernatants were poured off, cells washed and resuspended in supplemented DMEM for assays.

### 4.6 Flow cytometry

Cells isolated from spleen, lungs or peripheral blood were cultured with 2 µg/ml of two immunodominant peptides (Pepscan, Lelystad, The Netherlands), [SSTHEANTMAMMARDT] and [AGYAGTLQSLGAEIAV] of the TB10.4 protein, previously demonstrated to stimulate both CD4^+^ & CD8^+^ T cell responses (Kaveh & Hogarth, unpublished); 1 µg/ml anti-CD28 (BD Biosciences) and 10 µg/ml Brefeldin A (Sigma) for 16 h at 37 °C/5% CO_2_. Cells were washed at 300 g for 5 min and surface stained with combinations of CD62L-FITC, CD27-PerCP-Cy5.5, CD8-AF700, CD44-BV421, CD127-PE-Cy7, CD69-FITC, CCR7-BV421, live/dead-Zombie Aqua (all BioLegend) and CD4-APC-H7 (BD Biosciences). Cells were then washed, treated with BD Biosciences Cytofix/Cytoperm as per manufacturer’s instructions and stained intracellularly with combinations of IFN-γ-PE-Cy7, IL-2-APC (both eBioscience), IFN-γ-BV605 and TNF-α-BV605 (both BioLegend). Cells were washed again and analysed using an LSRFortessa™ analyser utilising a 532 nm laser for PE and PE-conjugate excitation with FACSDiva™ software (BD Biosciences). Final analysis was performed using FlowJo® software (Tree Star Inc.) on a minimum of 100,000 live lymphocytes (50,000 for peripheral blood).

### 4.7 Statistical analysis

All data were analysed using GraphPad Prism 7 statistical package (GraphPad, USA). When comparing two groups, an unpaired Student’s two-tailed *t*-test was performed. With three or more treatment groups the data were analysed by one-way ANOVA with appropriate multiple comparisons test as stated. Where two independent variables were compared, data were analysed by two-way ANOVA with appropriate multiple comparisons test as stated. For all data, * represents *P* < 0.05, ** represents *P* < 0.01, *** represents *P* < 0.001 and **** represents *P* < 0.0001.

## 5 Author contributions

PJH, HM and NCB conceived and designed the experiments. NCB, DAK and MCG performed the experiments. NCB analysed the data with assistance from DAK and ES and wrote the initial draft of the manuscript. PJH, HM, DAK and ES contributed to data interpretation and provided critical input to the manuscript.

## 6 Funding

This work was supported by the Department for Environment, Food and Rural Affairs under grant number SE3266 (to PJH) and the Wellcome Trust (Senior Clinical Research Fellowship held by HM).
