# Supplementary material for: Induction and maintenance of a phenotypically heterogeneous lung tissue-resident CD4+ T cell population following BCG immunisation
Source: Vaccine. 2018 Sep 5;36(37):5625–35. doi: 10.1016/j.vaccine.2018.07.035 (PMC6143486; doi:10.1016/j.vaccine.2018.07.035)
Supplement: Supplementary file 1 [file mmc1.pdf]

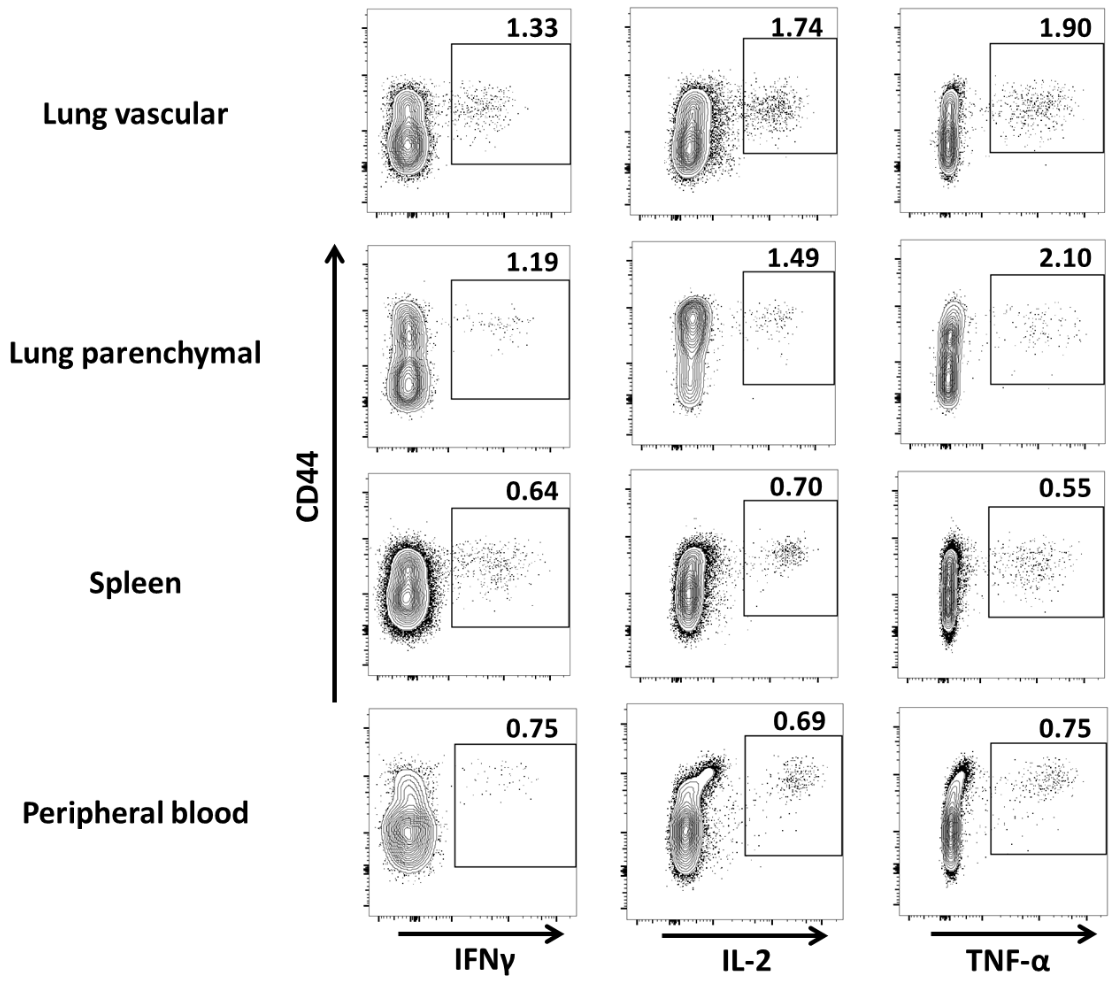

Supplementary Figure 1 Cytokine production by CD4$^+$ T cells in different tissue compartments. Following BCG immunisation, intravascular staining and ICS identified populations of antigen-specific (cytokine$^+$) CD4$^+$ T cells. Representative plots show IFN-γ, TNF-α and IL-2 production from CD4$^+$ T cells in the lung parenchyma, lung vasculature, spleen and peripheral blood of a mouse 5 weeks post-BCG vaccination.

Week 3

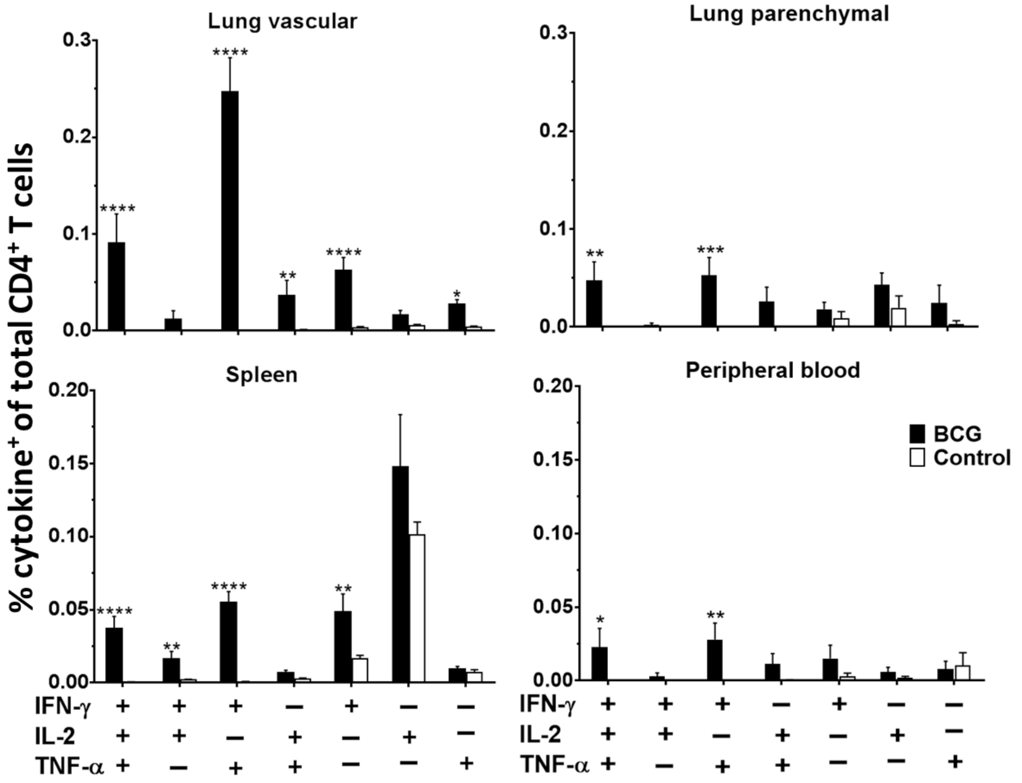

Lung vascular
Lung parenchymal
Spleen
Peripheral blood
% cytokine+ of total CD4+ T cells
BCG
Control
IFN-γ + + + − + − −
IL-2 + + − + − + −
TNF-α + − + + − − +

Week 6

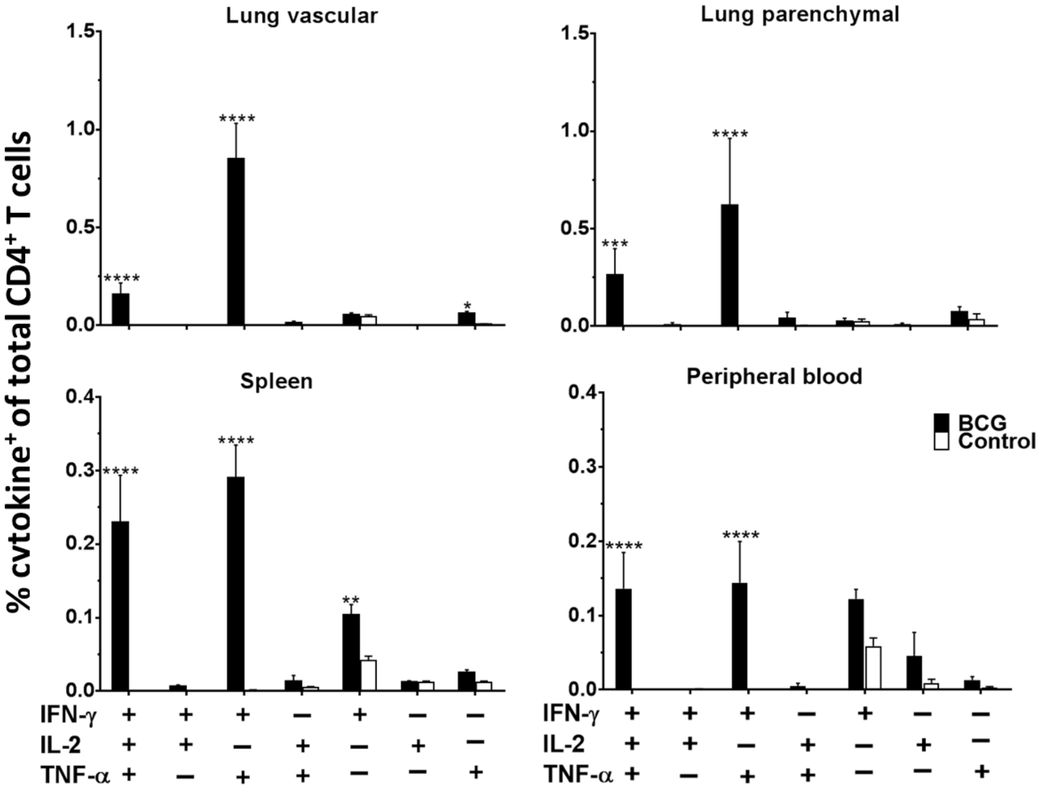

Lung vascular
Lung parenchymal
Spleen
Peripheral blood
% cytokine+ of total CD4+ T cells
BCG
Control
IFN-γ + + + − + − −
IL-2 + + − + − + −
TNF-α + − + + − − +

Week 12

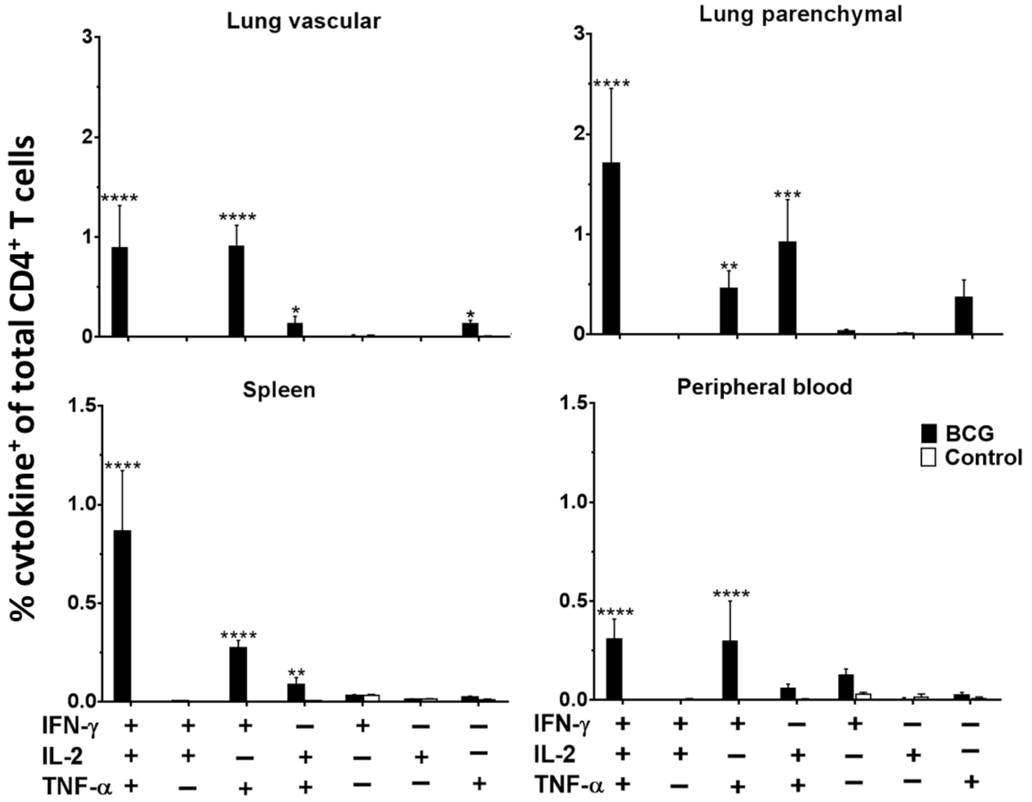

Week 26

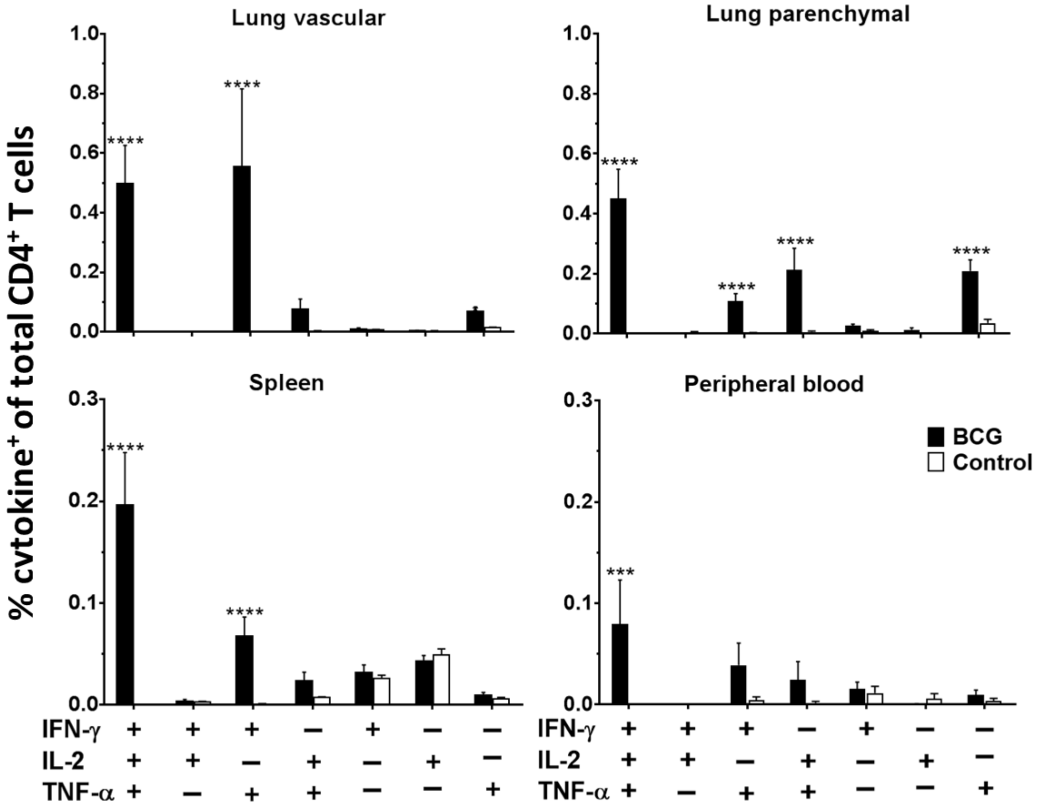

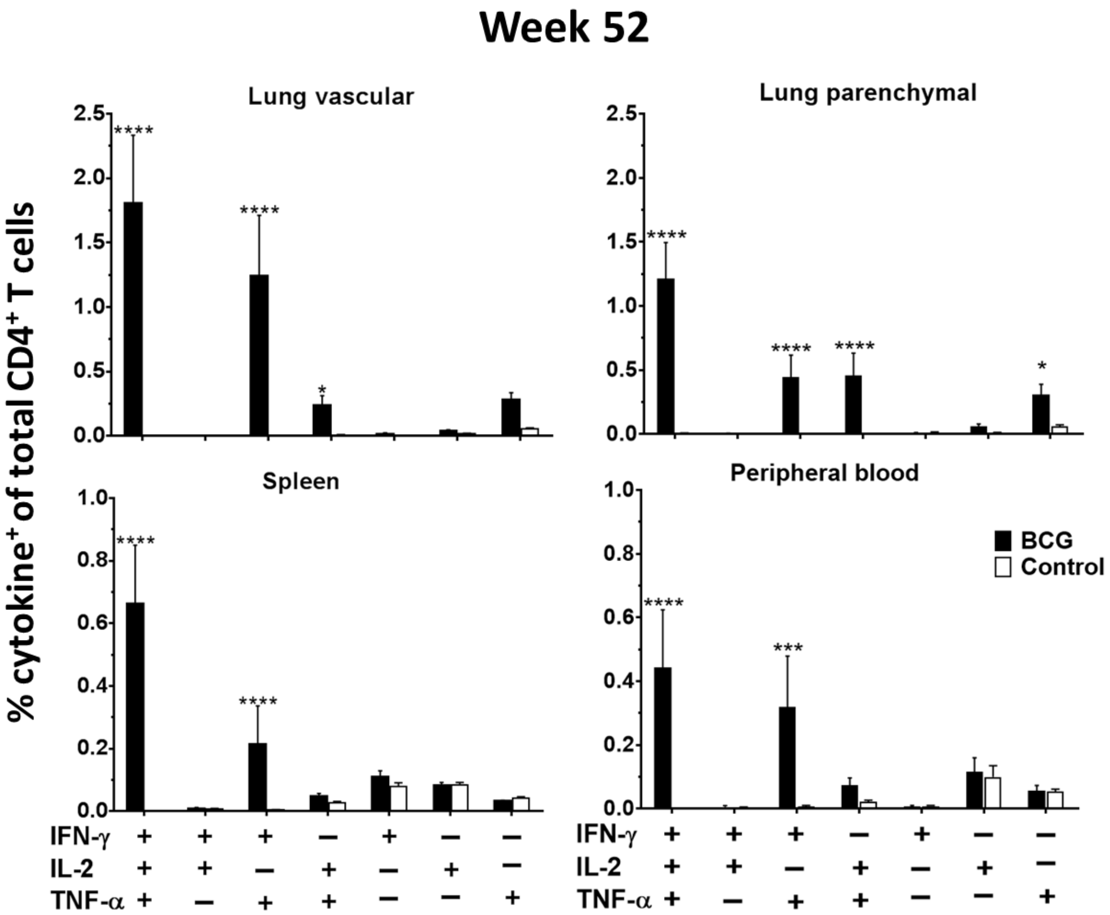

Supplementary Figure 2 Production of IFN-γ, IL-2 and TNF-α by CD4+ T cells post-BCG vaccination. Following BCG immunisation, intravascular staining and ICS identified populations of antigen-specific (cytokine+) CD4+ T cells in the lungs, spleen and peripheral blood producing IFN-γ, IL-2 or TNF-α alone or in combination. Graphs show frequencies of antigen-specific CD4+ T cells in all compartments as a % of the total CD4+ T cells in the same compartment at each time point. For all graphs, bars represent mean ± SEM (n=6). Two-way ANOVA with Sidak's post-test, comparing BCG and control, * P<0.05, ** P<0.01, *** P<0.001, **** P<0.0001.

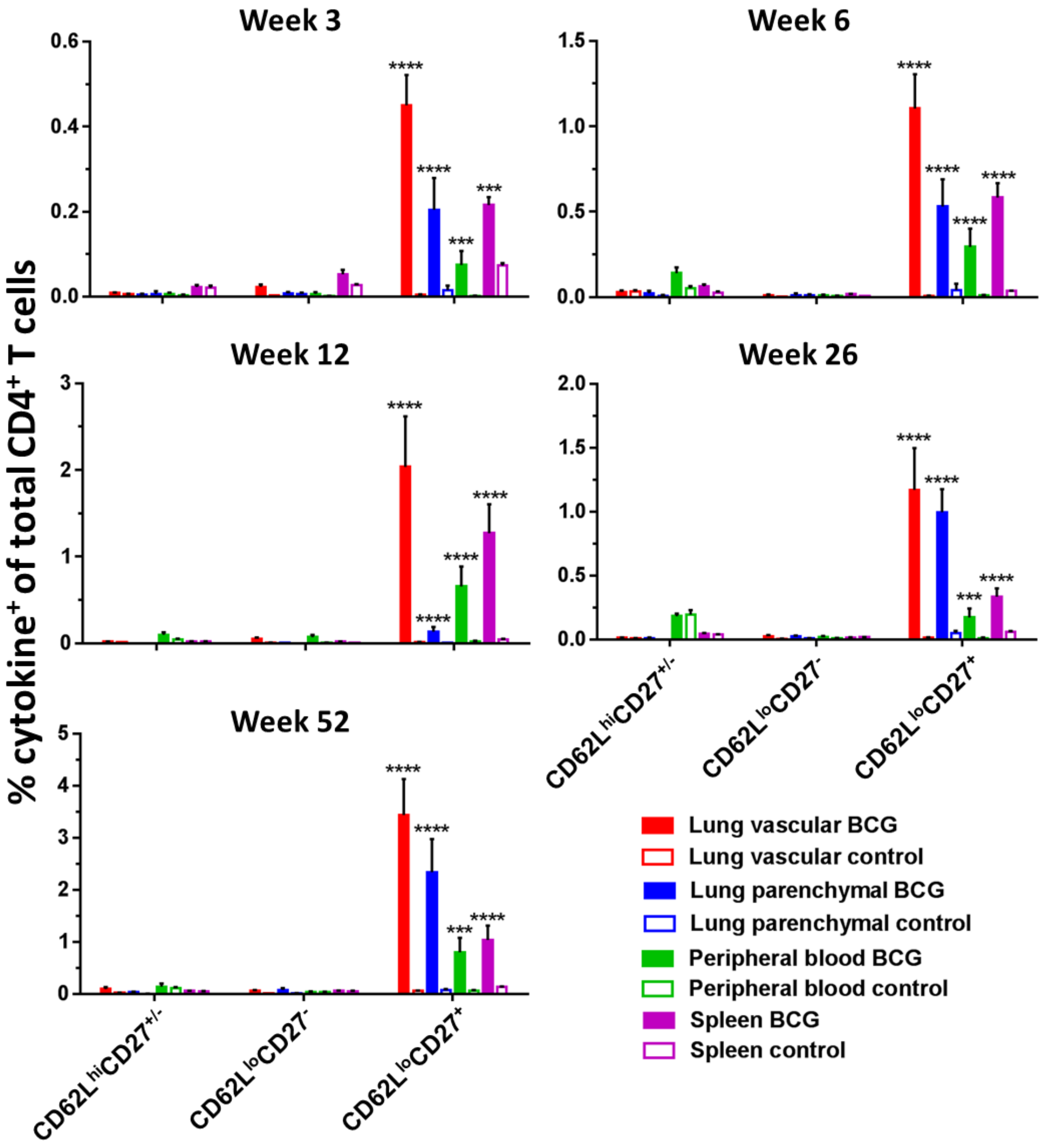

Supplementary Figure 3 BCG-induced antigen-specific CD4$^+$ T cells display an effector phenotype at all time points measured post-BCG vaccination. Following BCG immunisation, intravascular staining and ICS identified populations of antigen-specific (cytokine$^+$) CD4$^+$ T cells. Graphs show frequency of antigen-specific CD4$^+$ CD44^{hi} T cells in all compartments displaying combinations of CD62L and CD27 cell surface markers as a % of total CD4$^+$ T cells in that compartment. Bars represent mean ± SEM (*n*=6). Two-way ANOVA with Sidak's post-test, comparing BCG and control, *** *P*<0.001, **** *P*<0.0001.

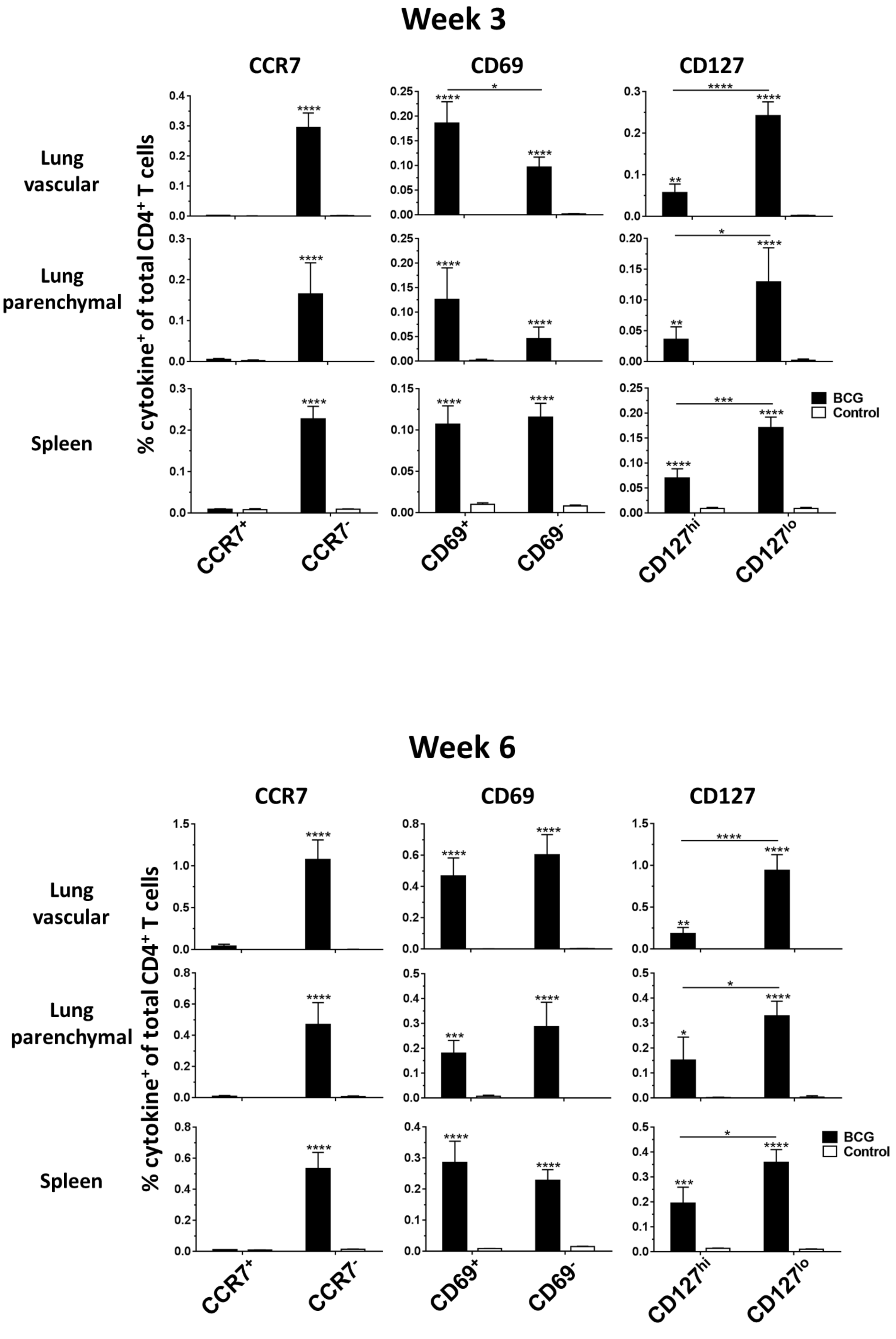
Week 3
CCR7
CD69
CD127
Lung vascular
Lung parenchymal
Spleen
% cytokine⁺ of total CD4⁺ T cells
BCG
Control
CCR7⁺
CCR7⁻
CD69⁺
CD69⁻
CD127hi
CD127lo
Week 6
CCR7
CD69
CD127
Lung vascular
Lung parenchymal
Spleen
% cytokine⁺ of total CD4⁺ T cells
BCG
Control
CCR7⁺
CCR7⁻
CD69⁺
CD69⁻
CD127hi
CD127lo

Week 12

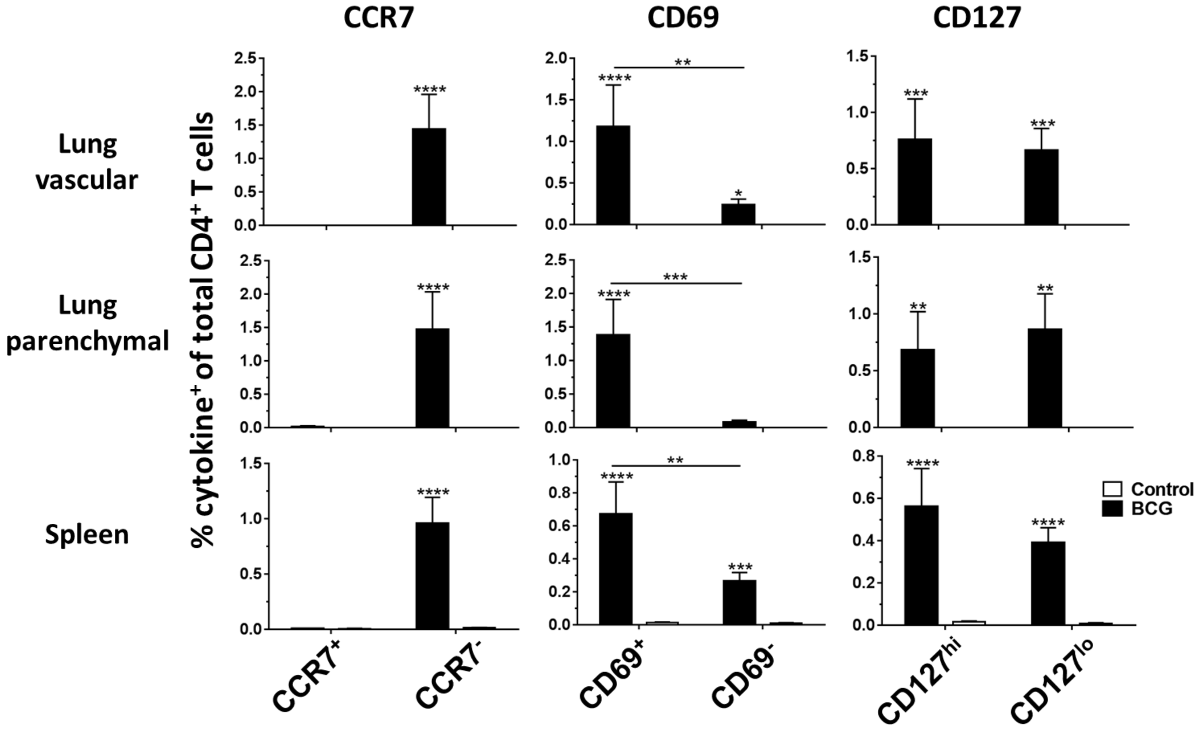

Week 26

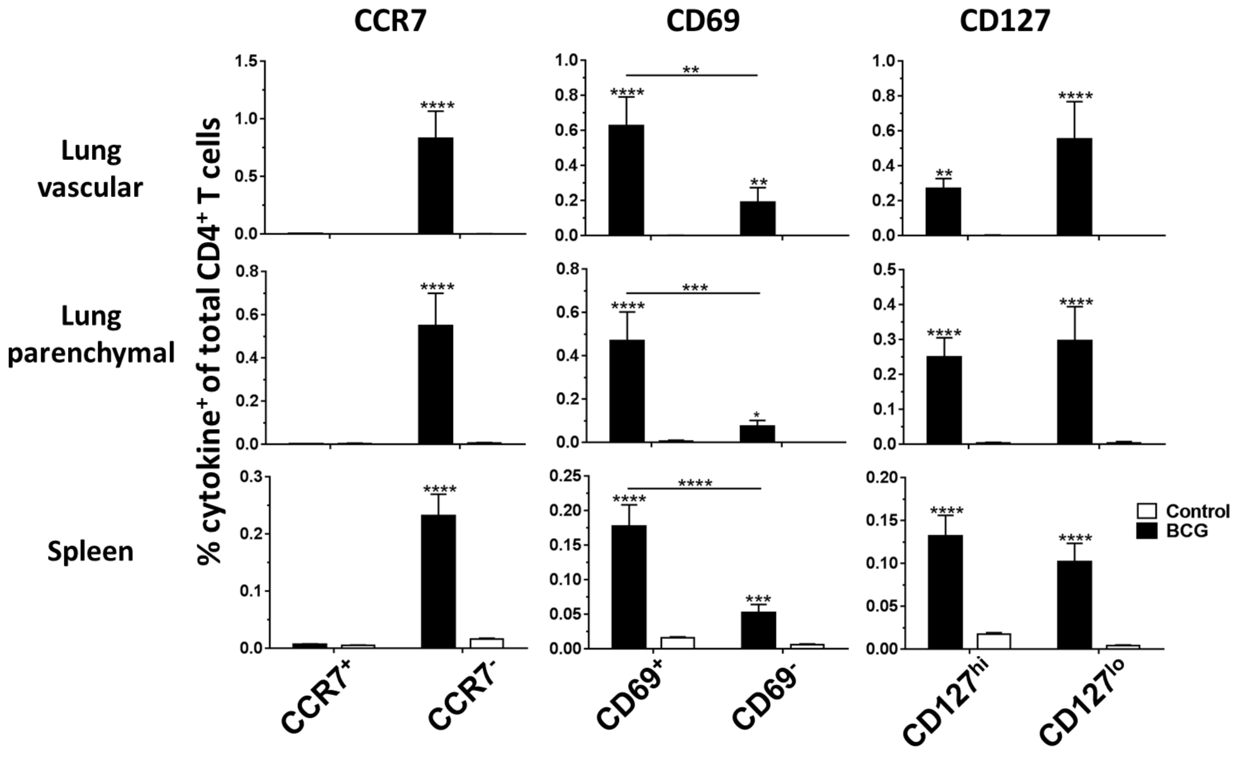

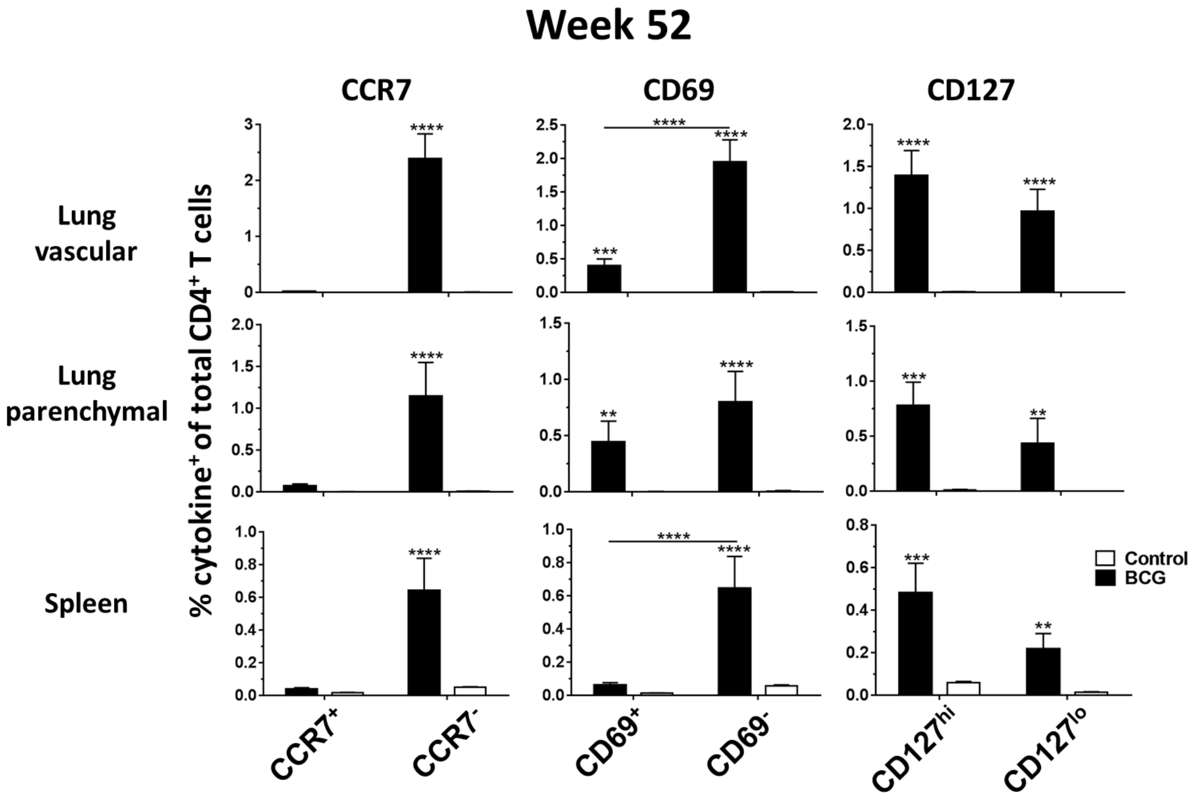

Supplementary Figure 4 BCG-induced antigen-specific CD4$^+$ T cells are CCR7$^-$ but heterogeneous for CD69 and CD127 at all time points measured post-BCG vaccination. Following BCG immunisation, intravascular staining and ICS identified populations of antigen-specific (cytokine$^+$) CD4$^+$ T cells. Graphs show frequencies of antigen-specific CD4$^+$ CD62L^{lo} T cells in all compartments displaying CCR7, CD69 and CD127 cell surface markers as a % of total CD4$^+$ T cells in that compartment. For all graphs, bars represent mean ± SEM (*n*=6). Two-way ANOVA with Sidak's post-test, comparing BCG with control and BCG with BCG, * $P<0.05$, ** $P<0.01$, *** $P<0.001$, **** $P<0.0001$.

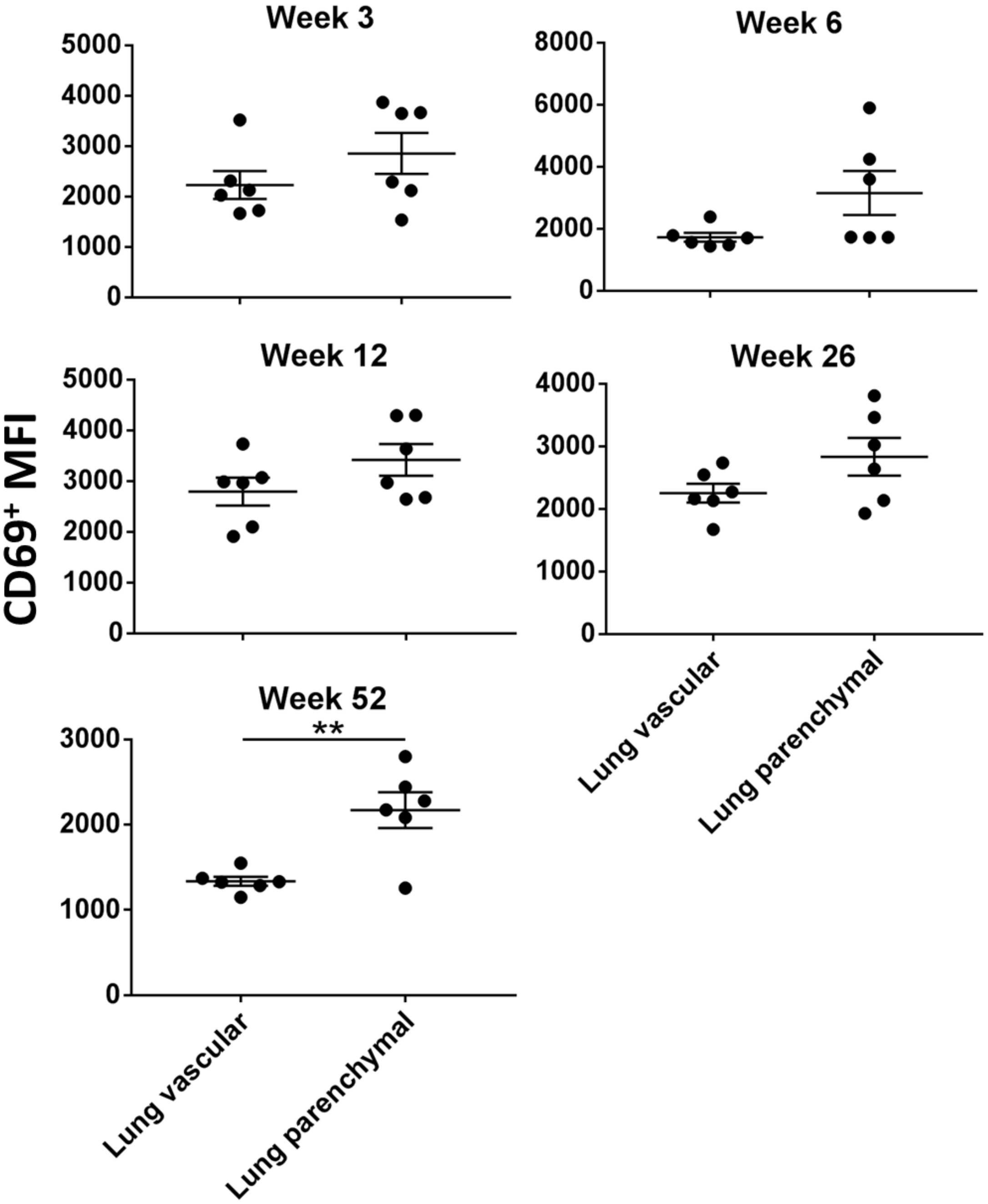

Supplementary Figure 5 Expression of CD69 on BCG-induced antigen-specific CD4⁺ T cells in the lung at all time points measured post-BCG vaccination. Following BCG immunisation, intravascular staining and ICS identified populations of antigen-specific (cytokine⁺) CD4⁺ T cells. Graphs show MFI of cytokine⁺ CD69⁺ populations in the lung parenchyma and lung vasculature. For all graphs, lines represent mean ± SEM (*n*=6). Unpaired two-tailed t-tests, ** *P*<0.01.
